# The mental wellbeing of female prisoners in Chile

**DOI:** 10.1186/s13104-023-06342-x

**Published:** 2023-05-15

**Authors:** Anne Aboaja, Douglas Blackwood, Rubén Alvarado, Liz Grant

**Affiliations:** 1 Tees, Esk and Wear Valleys NHS Foundation Trust, Darlington, UK; 2 University of Edinburgh, Edinburgh, UK; 3 University of York, York, UK; 4 University of Chile, Santiago, Chile; 5 University of Valparaíso, Valparaíso, Chile

**Keywords:** Mental wellbeing, Latin America, Prison, Women, Forensic psychiatry, WEMWBS, Global mental health

## Abstract

**Objective:**

To measure and understand mental wellbeing among women prisoners in Chile, as part of a larger study.

**Result:**

Sixty-eight sentenced prisoners in a women’s prison participated in a survey, giving a response rate of 56.7%. Using the Warwick-Edinburgh Mental Wellbeing Scale (WEMWBS), the mean wellbeing score of participants was 53.77 out of maximum score of 70. Whilst 90% of the 68 women felt useful at least some of the time, 25% rarely felt relaxed, close to others or able to make up their own minds about things. Data generated from two focus groups attended by six women offered explanations for survey findings. Thematic analysis identified stress and loss of autonomy due to the prison regime as factors which negatively affect mental wellbeing. Interestingly, whilst offering prisoners an opportunity to feel useful, work was identified as a source of stress. Interpersonal factors linked to a lack of safe friendships within the prison and little contact with family had an adverse impact on mental wellbeing. The routine measurement of mental wellbeing among prisoners using the WEMWBS is recommended in Chile and other Latin American countries to identify the impact of policies, regimes, healthcare systems and programmes on mental health and wellbeing.

## Introduction

Mental disorders have been extensively studied among international prisoner populations where the prevalence of mental disorder is higher than that of the general population [1, 2]. In contrast, the distinct but significantly linked concept of mental wellbeing [3–5] has not been widely studied among prisoners, despite evidence that mental wellbeing predicts the onset of [6] and recovery from mental disorder [7]. A study in Scotland showed poorer mental wellbeing among female prisoners than women in the general population [8]. Low wellbeing scores among the women were partly explained by a high proportion reporting “not feeling good about [themselves]” and “not feeling relaxed” [9]. However, there is a lack of international data from prison mental health studies that can be compared with these findings.

There are almost 40,000 prisoners in Chile where women are imprisoned at a rate twice as high (20.9%) as the global average (9.9%), and represent 7.5% of the total prison population [10]. The prevalence of common mental disorders in Chile is higher among prisoners than the general population [11]. In Chile, the mental wellbeing has been measured in the general population but not among prisoners [12]. Given the high female prison population in Chile and the general need for international studies to better understand mental health wellbeing in female prisons, building on previous work undertaken in the country, this study aims to measure and understand the mental wellbeing of female prisoners in Chile.

## Main text

### Design and methods

The Chilean Ministry of Justice and the ethics committees of the Centre for Population Health Sciences at the University of Edinburgh and the Faculty of Medicine at the University of Chile (Proy. 0432015/0442015) approved the study. It was part of a larger study of mental health and spirituality among female prisoners. We used an explanatory sequential mixed methods design [13] comprising a cross-sectional survey and focus groups.

#### Sample

Participants were recruited from a prison for sentenced women in Chile. On the first day of the study the prison had a total population of 800. The prison has ten sections including one for high-risk prisoners, one supporting women with children up to the age of two years, one offering a full-time paid work programme, two managed by religious institutions, and one with a semi-open regime for women undertaking part-time rehabilitation projects in the community. The prison health centre workforce includes two primary care physicians and general nurses who provide all on-site mental health care. There are no specialist mental health professionals in the prison. Prisoners requiring tertiary mental health care are transferred to a prison hospital at another site. Women who were not residing in the high-risk section [11], were randomly selected and invited to participate in the study.

#### Data collection

Consenting participants were interviewed in Spanish and completed a survey about demographics, heath and religious beliefs. The primary mental health outcome was mental wellbeing measured using the Chilean Spanish version [12] of the Warwick-Edinburgh Mental Wellbeing Scale (WEMWBS) [14, 15]. The WEMWBS comprises 14 statements of mental wellbeing domains for which participants indicate the frequency with which they have experienced each domain over the preceding two weeks. A total WEMWBS score of 14 represents the lowest level of mental wellbeing; a score of 70 reflects the highest level of mental wellbeing.

Eighteen months later, prisoners who had participated in the larger study and were still incarcerated were invited to participate in focus groups to discuss the survey topic and findings. The topic guide was designed to elicit participants’ views on mental health (which conceptually included mental disorder and mental wellbeing), beliefs about factors affecting prisoner mental health, and responses to the findings from the cross-sectional survey. Focus groups were led in Spanish by two facilitators and audio-recorded.

#### Analytical methods

To ensure the study would be adequately powered [14] to accurately estimate the mental wellbeing of the study population, after anticipating non-participation rates of at least 60% [11, 16], a power calculation was undertaken using the formula [((Z_1-α_)^2^(SD)^2^)/d^2^]. Missing quantitative data were managed through complete case analyses. Descriptive analyses were undertaken of quantitative data. Focus group audio recordings were transcribed in Spanish. Data were coded and analysed thematically in frameworks within a series of spreadsheets using *a priori* codes such “difference in mental wellbeing of women in prison and outside of prison” which was based on the findings from the cross-sectional survey and the literature, and codes emerging *de novo* from the data such as “loneliness” [17–19]. Dynamic equivalence guided the translation of selected quotations into English.

#### Results: survey findings

From the total prison population (n = 800), excluding 18 high-risk women [11], 120 women were randomly selected through a computer-generated randomisation programme to be surveyed on demographics, health and beliefs. Of these, sixty-eight (56.7%) women participated in the cross-sectional survey. Reasons for non-participation included: ill-health, lack of capacity, high-risk, and work commitments.

Of the 68 participants surveyed, 59 (86.7%) participants provided complete data for the WEWMBS. This was a sufficient number based on the power calculation (n = 37). The mean age was 39.4 years. Table 1 shows that thirty-three (56.9%) participants were single and over two-thirds (71.2%) had children under the age of 19. Just over half (n = 31, 53.4%) of the women were unemployed prior to incarceration. Fifty-one (89.5%) women identified with Christianity. Religion and spirituality were important to most women (n = 49, 86.0%).

Most (n = 39, 67.2%) women reported a drug-related index offence, whilst four (6.9%) stated they were serving a sentence for a violent offence. All prison sections were represented by those who participated in the study with the exception of the mother and baby prison section. Over half of all participants were from either the work or evangelical Christian sections of the prison.

Forty-five women (77.6%) shared a room with at least nine other prisoners. Two-thirds of the women smoked regularly in prison. Two (3.4%) women reported drinking alcohol in prison whilst 21 (35.6%) admitted to using drugs in prison.

**Table 1:** Characteristics of female prisoners who completed the WEMWBS survey

Variable		N = 59	%
Age (years)*	18–24 25–34 35–44 45–54 55–64	3 20 17 11 6	5.2 35.1 29.8 19.3 10.5
	Mean age = 39.4 (SD 10.8)		
Marital status**	Single Married/Serious relationship Separated/Divorced/Widowed	33 12 13	56.9 20.7 22.4
Number of children < 19 years	0 1–3 4–6 7–9	17 33 8 1	28.8 55.9 13.6 1.7
Nationality	Chilean Other	58 1	98.3 1.7
Education level	Primary Secondary Technical college	34 23 2	57.6 39.0 3.4
Pre-incarceration employment status**	Unemployed Self-employed Employed	31 16 11	53.4 27.6 19.0
Religious affiliation	Christianity Other (belief in God)	51 8	89.5 10.5
Level of personal importance of religion and spirituality	High Low	49 10	86.0 14.0
Primary index offence**	Violent/sexual Acquisitive Drug-related	4 15 39	6.9 25.9 67.2
Number of other prisoners with whom they shared a room**	1–3 4–6 7–9 > 9	1 7 5 45	1.7 12.1 8.6 77.6
Number of cigarettes smoked daily in prison	0 1–5 6–10 11–20 21–30	18 21 7 12 1	30.5 35.6 11.9 20.3 1.7
Alcohol consumption	Never Before imprisonment but not in prison In prison	17 40 2	28.8 67.8 3.4
Illicit drug use in prison	Never Before imprisonment but not in prison In prison	22 16 21	37.3 27.1 35.6

*Data from 57 participants

**Data from 58 participants

The mean WEWMBS score was 53.77 (SD 11.05). Table 2 shows that at least 90% of participants had for at least some of the time during the preceding two weeks experienced at least one of the following: feeling useful, thinking clearly, being able to make up their own minds about things, and being interested in new things. In contrast, at least 25% of participants had during the same time period rarely or never experienced: feeling relaxed, having energy to spare or feeling close to other people.

**Table 2:** Mental wellbeing (WEMWBS) of participants

WEMWBS item	Dimension of mental wellbeing experience during the previous two weeks	Chile (present study)				
		None of the time/ Rarely		Some of the time/ Often/All the time		Total
		n	%	n	%	N
**1**	**Feeling optimistic about the future**	14	24.14	45	77.59	59
**2**	**Feeling useful**	4	6.90	55	94.83	59
**3**	**Feeling relaxed**	18	31.03	41	70.69	59
**4**	**Feeling interested in other people**	8	13.79	51	87.93	59
**5**	**Had energy to spare**	15	25.86	44	75.86	59
**6**	**Dealing with problems well**	7	12.07	52	89.66	59
**7**	**Thinking clearly**	5	8.62	54	93.10	59
**8**	**Feeling good about myself**	9	15.52	50	86.21	59
**9**	**Feeling close to other people**	17	29.31	42	72.41	59
**10**	**Feeling confident**	12	20.69	47	81.03	59
**11**	**Able to make up my own mind about things**	5	8.62	54	93.10	59
**12**	**Feeling loved**	8	13.79	51	87.93	59
**13**	**Interested in new things**	3	5.17	56	96.55	59
**14**	**Feeling cheerful**	11	18.97	48	82.76	59

#### Results: focus group findings

Six women who had completed the cross-sectional survey in the larger study participated in two focus groups. The remaining women did not attend for the following reasons: they were no longer in the prison, they declined to join the qualitative part of the study or they consented to the study but failed to attend the focus group. Reasons given by women who chose not to consent to the focus groups included: not wishing to be in a group, having a dislike of talking to people in the prison, lack of time due to work commitments, wishing to speak only to God, dissatisfaction with the prison health service, and believing that things would not change by attending a focus group. One of the focus group participants pointed out that she had considered not attending the focus group because she would face financial penalties for being absent from her work placement inside the prison. Of the six women who attended the focus groups, all of whom were within the 18–64 age range, three (50%) were single, four (67%) had not progressed beyond primary education, three (50%) had a drug-related primary index offence, five (83%) were affiliated to Christianity, and all (100%) considered religion and spirituality to be of highest personal importance. Three (50%) had a WEMWBS score between 21 and 30, whilst the remaining women had a WEMWBS score between 51 and 60. They were representative of the surveyed group in terms of demographics and mental wellbeing scores. Table 3 shows the relevant themes that emerged from the focus groups. Stress and loss of autonomy were identified as prison regime themes that mapped onto three WEMWBS items. Participants identified two prison-related interpersonal themes (a lack of trusting relationships and reduced family contact) that linked to an additional two WEMWBS items.

**Table 3:** Themes arising from the focus groups on the mental wellbeing of female prisoners

Category	Theme	Illustrative quotes from focus group participants	WEMWBS item
**Prison regime**	Stress	“We are stressed through the work, that they require so much from us” (FG1) “Here there are prison officers who humiliate you and ill-treat you” FG1 “The prison stresses” (FG1) “It is a depression to be depending on the officers who suddenly humiliate you, that suddenly mistreat you” (FG2) “It make me nervous.“ (FG1)	Item 3 - Do not feel relaxed
			Item 5 - Do not have energy to spare
	Loss of autonomy	“One is not free here to go to bed in the daytime, the bedrooms are closed…Here you cannot make your own decisions about something, about going to eat in peace because the prison officer arrives and tells us ‘Stand up!” (FG1) “It is that the food here is bad” (FG1) “Here where I am, there is no telephone, only public telephones nothing else,” (FG2) “What annoys me most in here is that we are prisoners and because we are prisoners it is like we were sentenced not to speak. we pass as being one animal more” (FG1)	Item 11 – Are not able to make up their own minds about things
**Inter-personal**	Lack of trusting peer relationships in prison	“Here in the prison, there are no friends.” (FG2) “Here one feels alone…you don’t have anyone to tell things to because sometimes you trust and afterwards, they know it, you keep everything inside sometimes, huge pains, you have to keep them inside, no more, you can’t tell it because they’ll laugh at you, " (FG2) “There are no friends here to start with. There are acquaintances and you can’t tell your things to another person because that person is going to talk to another person and in the end everyone knows and it is not something you want them to know because they are personal things and sometimes you need to let off steam with someone but you can’t for the same reasons…I’ve drifted away from many people for these reasons” (FG1) “The rule…is that here…one cannot (this also gives you depression) express oneself with anyone, tell them…things, because in whatever moment, whatever fight, they will shout it…in front of others, and that makes you feel bad, worse” (FG2) “What it is, is that here there is a lot of evil and jealousy…egoism and egocentricism” (FG1) “No, here there is much jealousy…they can be with you for money or for the things that they [your family???] bring you” (FG2) “it is difficult to find friends” (FG2) “Not having anyone, suddenly being alone, one believes one is alone buy you are not alone” FG2 “Of course I feel alone, nobody helps me” (FG2)	Item 9 - Do not feel close to others
	Difficulty maintaining contact with family outside of prison	“There are girls who get into drugs because they are alone, or they are left alone, and they [the family] don’t come to see them.” (FG2) “Because they are locked up…because they are alone in these places” (FG2) Mothers who have children and they don’t come to see them, that contributes much to depression. (FG1) “I haven’t seen my children for 8 months or my mother or anyone from my family because I have no visits because if one sometimes commits many errors but the family always reminds you of it. My mother reminds me of it always. She won’t let me see my children. She doesn’t bring them to me and that’s why I am ill [cries uncontrollably]” (FG1)	Item 9 - Do not feel close to others
		“We are so lacking in affection” (FG1) “Lack of love” (FG2) “lack of affection, suddenly one thinks no one loves here” (FG1) “lack of love, of affection” (FG1)	Item 12 – Do not feel loved

Most participants in both groups held the view that increased levels of religiosity and spirituality contribute to increased levels of mental wellbeing that might mitigate the prison themes reported in Table 3:

*“The gospel, let’s go to the spirituality of the gospel, well, to be in something that helps you because God helps. God, you speak to him and he listens. When you have pain, when you are tired, when you are distressed, he hears you. He never leaves you alone…one wants to get alongside him, him, no, on the contrary, he is always here with us.”* (FG1)

## Discussion

This is the first study to apply a culturally validated version of the WEMWBS to measure mental wellbeing in a prison population in Chile. This study estimates a mean WEMWBS score of female prisoners in Chile of 53.7. Notably, most female prisoners experience the 14 positive domains of mental wellbeing at least some of the time. However, some women viewed prison as an institution which exposes them to factors that can have a negative impact on mental wellbeing. Notable findings that women were less likely to feel relaxed and have energy to spare were explained by reports of stressful work demands of the prison regime. Similarly, the lack of friendships amongst fellow prisoners and the challenges in maintaining family contact whilst in prison were given as reasons why women were less likely to feel close to other people.

When compared to the WEMWBS mean of 56.6 in the general female population in Chile [12], our results are consistent with that of the UK literature reporting lower WEMWBS scores among prisoners than the general population [8]. Lack of activity and stimulation in prison contributes to increased stress levels among British prisoners who, consistent with the participants of the present study, also report difficulties in maintaining contact with family [20] and finding peers inside the prison who can be trusted [21]. These findings are underpinned by the social psychology understanding of belonging as a fundamental human need without which individuals can experience poor mental wellbeing [22]. This is particularly problematic among prisoners who may experience difficulty in trusting others which then hinders their ability to experience social connectedness [23]. Health-promoting social connections that will fulfil the need for belonging must be mutually positive and caring [22], unlike those relationships of disloyalty, jealousy and secondary gain described by female prisoners in the present study. Furthermore, the lack of trust leading prisoners to refrain from speaking about personal matters with others in prison may have had an impact on the decision of some surveyed women who chose not to join a focus group. Whilst we did not undertake analyses to identify associations between WEMWBS and several demographic factors, there is evidence that in the general population in the UK, a higher WEMWBS score is associated with being married, attaining a higher level of education, being employed and being middle-aged [14]. The mean age, low educational level, high unemployment level prior to incarceration and increased single status could partly explain the poorer mental wellbeing found among female prisoners compared to the general population.

The WEMWBS has been used among prisoners in Africa [24], Asia [25] and Europe [8], but prior to the present study, not in Latin America. We acknowledge the limitations in comparing findings from our study with similar data arising from other countries due to differences in population (for example, length of sentence being served), prison regime (for example, family visiting policies, prison occupancy and practices that enable older children reside with their incarcerated mothers) and wider systemic factors (e.g., the presence of legislation which diverts prisoners with mental illnesses away from the criminal justice system into the health system). Worldwide, comparable WEMWBS data for female prisoners is both scarce and small. Studies are often not designed to identify any gender differences. Equivalent evidence from a large (n = 240) mixed-gender prisoner study in Zambia included WEBWBS data from only six female prisoners [24] and a study of 198 prisoners in India collected WEMWBS data from 17 women [25]. Neither study stratified data by gender to measure the WEMWBS of female prisoners. We therefore rely on comparison data from the largest WEMWBS study of female prisoners to date undertaken in Scotland.

We found that, among a modest sample of 59 randomly selected female prisoners from a single site in Chile, the mental wellbeing was estimated to be higher (mean WEMWBS = 53.7) than that reported among a convenience sample of 152 women in five Scottish prisons (mean WEMWBS = 41.4). Across each WEMWBS domain, female prisoners in Chile reported higher levels of mental wellbeing than did their Scottish counterparts [8]. The difference was most marked for the WEMWBS domains of feeling useful and good about oneself.

A possible explanation is that the Scottish group comprised not only sentenced women, but also those on remand who were therefore more likely to have had poorer mental wellbeing [8]. Factors unique to the Chilean setting may also explain the higher mental wellbeing. Firstly, among the female prisoners in Chile, there was a high level of religiosity and spirituality (predominantly Christian affiliation) which has in other countries been linked to fewer depressive symptoms [26]. In contrast, over half (57%) of Scottish female prisoners at a similar time did not identify with a religion [27]. The complex association between religiosity and spirituality, and mental wellbeing among prisoners [28] which was mentioned by participants is an important topic for further study in the Chilean context. Secondly, compared to the predominantly single-cell occupancy prison environment in Scotland, in Chile most women lived in conditions of multiple occupancy. Although this did not seem to provide an environment in which trusting relationships between prisoners could be fostered, it may have offered protection against the risk of suicide which has been widely reported [29]. Thirdly, whilst on the one hand high levels of work activity in Chile reportedly added to the stress experienced, on the other hand it may have alleviated or prevented feelings of useless (WEMWBS item 2).

When compared to the limited international data, the observed differences in mental wellbeing call for further exploration. First, more data is needed about the mental wellbeing of female prisoners in high, middle and low income countries. Where mixed-gender data are collected among prisoners, analyses should be undertaken by gender to identify and understand any differences between men and women. A mixed methods comparative study of Chile and Scotland would help to answer the key global health question arising from this study: is the mental wellbeing of female prisoners better in Chile than in Scotland and if so, why? A larger quantitative study in male and female prisons in Chile would not only provide an estimate of the mental wellbeing among male prisoners, but also identify predictors of poor mental wellbeing which could further inform prison policy and practice.

The present findings demonstrate that the WEMWBS could be used routinely as part of a national prison health survey in Chile and even in other parts of Latin America. Measuring changes in the WEMWBS of prisons will highlight trends and the potential impact of significant changes such as prison mental healthcare provision, policies, programmes and regimes on the mental wellbeing of prisoners. A whole-system approach involving policy makers, prisoner officers, employment providers, social workers, family link workers, health professionals and chaplains working in collaboration with prisoners is needed to address the prison regime and interpersonal factors that could improve the mental health of female prisoners. For example, prison policies that promote increased choice of daytime activities for prisoners are likely to improve mental wellbeing [30] and there may be added mental wellbeing benefits from of occupational therapy-based activities [31]. Programmes aimed at facilitating family visits with children for women in prison [32] would address not only the poor mental wellbeing arising from lack of family contact, but also reduce the risk of suicide during incarceration [33]. Finally, interventions delivered by prison officers, social workers or health professionals to increase the mental wellbeing of female prisoners in Chile are more likely to be acceptable if they are culturally sensitive [34] and in this population religious and spiritual factors should be considered.

## Limitations

Whilst random sampling reduced sampling bias, the exclusion of high risk prisoners is likely to have reduced the representativeness of the overall prison population. Increased validity was achieved through the use of a version of the WEMWBS that had been psychometrically tested and adapted for use in Chile. However, the Chilean version had been validated in the general population which had a higher level of education (60% had completed secondary education) than the studied prisoner population of which almost two-thirds had not completed secondary education. The passage of time between the quantitative data collection and the focus group may have not only affected the number of focus group participants due to some having been released from prison, but also weakened the link between the quantitative and qualitative findings if prison policies and regimes changes during that period. The qualitative findings of the present study, whilst cautiously interpreted due to the small sample size, offer tentative explanations of the mental wellbeing profile of female prisoners in Chile.

## Data Availability

The datasets generated and analysed during the current study are not publicly available due to the sensitive nature of the data regarding offences, health information and the prison system, but are available from the corresponding author on reasonable request and with permission of the Ministry of Justice in Chile.

## List of Abbreviations

WEMWBS: Warwick-Edinburgh Mental Wellbeing Scale
